# Investigating the effects of beta-blockers on circadian heart rhythm using heart rate variability in ischemic heart disease with preserved ejection fraction

**DOI:** 10.1038/s41598-023-32963-0

**Published:** 2023-04-10

**Authors:** Shiza Saleem, Ahsan H. Khandoker, Mohanad Alkhodari, Leontios J. Hadjileontiadis, Herbert F. Jelinek

**Affiliations:** 1 Department of Biomedical Engineering, Khalifa University, 127788 Abu Dhabi, United Arab Emirates; 2 Healthcare Engineering Innovation Center, Khalifa University, 127788 Abu Dhabi, United Arab Emirates; 3 Biotechnology Center, Khalifa University, 127788 Abu Dhabi, United Arab Emirates; 4 Cardiovascular Clinical Research Facility, Radcliffe Department of Medicine, University of Oxford, Oxford, UK

**Keywords:** Heart failure, Predictive markers

## Abstract

Heart failure is characterized by sympathetic activation and parasympathetic withdrawal leading to an abnormal autonomic modulation. Beta-blockers (BB) inhibit overstimulation of the sympathetic system and are indicated in heart failure patients with reduced ejection fraction. However, the effect of beta-blocker therapy on heart failure with preserved ejection fraction (HFpEF) is unclear. ECGs of 73 patients with HFpEF > 55% were recruited. There were 56 patients in the BB group and 17 patients in the without BB (NBB) group. The HRV analysis was performed for the 24-h period using a window size of 1,4 and 8-h. HRV measures between day and night for both the groups were also compared. Percentage change in the BB group relative to the NBB group was used as a measure of difference. RMSSD (13.27%), pNN50 (2.44%), HF power (44.25%) and LF power (13.53%) showed an increase in the BB group relative to the NBB group during the day and were statistically significant between the two groups for periods associated with high cardiac risk during the morning hours. LF:HF ratio showed a decrease of 3.59% during the day. The relative increase in vagal modulated RMSSD, pNN50 and HF power with a decrease in LF:HF ratio show an improvement in the parasympathetic tone and an overall decreased risk of a cardiac event especially during the morning hours that is characterized by a sympathetic surge.

## Introduction

One of the first meta-analysis of use of beta-blockers (BBs) for heart failure (HF) with preserved left ventricular ejection fraction (HFpEF) included twelve studies included 21,206 patients and concluded that use of BBs may reduce all-cause mortality but not the number of hospitalizations^1^. A poor prognosis is associated with the number of hospitalizations in patients with HFpEF, which is similar to those with a reduced ejection fraction. With over 50% of patients falling into the HFpEF, treatment outcomes need to be improved based on a more comprehensive understanding of the pathophysiology and treatment effectiveness for heart failure. HFpEF is characterised by an abnormal diastolic function due to increased ventricular stiffness accompanied by possible comorbidities including atrial fibrillation, ischemic heart disease, hypertension, and renal disease leading to an approximate 8–12% annual mortality rate of the approximate 26 million people with HFpEF world-wide and increasing with an aging population^2^. Alternative, non-invasive and individualised screening is an option using wearable technology and heart rate variability analysis^3,4^.

Intrinsic and extrinsic regulation of the heart rhythm and force of contraction are important attributes of cardiac function and are compromised in heart failure. The abnormal autonomic nervous system (ANS) modulation of cardiac rhythm is associated with a decrease in carotid baroreceptor response, which leads to an increase in sympathetic nervous activity^5^ but both sympathetic activation and parasympathetic withdrawal are involved in the changing cardiac rhythm in early asymptomatic heart failure, with the compensatory sympathetic effect eventually leading to negative outcomes with disease progression^6,7^.

Beta-blocker (BB) therapy is based on the premise that sympathetic modulation needs to be reduced in heart failure and is therefore primarily prescribed for patients with a reduced ejection fraction (HFrEF) due to the overactive sympathetic activation. Several studies have shown the benefits of BBs on reducing hospitalizations and improving survival regardless of the BB used including the Study of the Effects of Nebivolol Intervention on Outcomes and Re-hospitalization in Seniors with Heart Failure (SENIORS) and Carvedilol Prospective Randomized Cumulative Survival (COPERNICUS)^8–11^.

However other studies including the Carvedilol Or Metoprolol European Trial (COMET) have indicated that BBs may have specific effects and should be selected carefully based on patient presenting signs and symptoms^12^. BB therapy was commenced in patients with HFpEF on the premise that BBs are efficacious in patients with impaired LVEF (< 40%) as they lower cardiac muscle work, reduce heart rate and therefore oxygen demand^13^. No clear guidelines however have been established for BB use which have varying activity and function. Depending on the medication BBs have adrenergic β-receptors selectivity, adjunctive effects on α-receptors, as well as effects on oxidative stress and inflammation.

Diverse pharmacokinetics, bioavailability and efficacy of different BB drugs has shown a heterogeneous patient response^13–16^. Pharmacotherapy is also not restricted in patients with heart failure and can include either as single medication or combined renin–angiotensin–aldosterone system antagonists, organic nitrates, digoxin and phosphodiesterase-5 inhibitors. Current European guidelines recommend commencing with angiotensin converting enzyme inhibitors (ACEIs) and BBs when HFrEF is diagnosed^10^. Less clear is BB pharmacotherapy for HFpEF where no data has been reported that indicates reduction in mortality and treatments effective for patients with HFrEF are not necessarily appropriate for HFpEF^17,18^. This is more than likely due to HFpEF being a heterogenous syndrome requiring precision medicine and individual patient targeted intervention including not only consideration of geographic and ethnic differences but the sympathovagal contribution to cardiac arrhythmogenesis in response to BBs^19,20^.

### Heart failure, beta-blockers and heart rate variability

Heart failure with preserved ejection fraction is becoming increasingly more common and is associated with sudden cardiac death most often due to ventricular arrhythmias^21–23^. Investigating cardiac rhythm utilizing heart rate variability (HRV) as an indicator has been shown to be a robust analytical method for identifying significant differences in cardiac rhythm associated with CVD, kidney disease as well as multiorgan disease such as diabetes and other pathology including depression^4,24–27^. Common heart rate variability (HRV) analysis includes short-time recordings between 24-h to 5 min. Holter 24-h recordings are most often analysed using cosinor analysis based on the HRV features^28–31^.

Decreased HRV is an independent risk factor for mortality following myocardial infarction patients with heart failure^32^. Use of BBs affects linear and nonlinear HRV features derived from ECG or heart rate recordings in patients with HFrEF using a diverse set of HRV features including Poincaré analysis, symbolic dynamics, multiscale entropy and detrended fluctuation analysis and providing a possible tool for risk stratification^33,34^. An earlier study of 24-h Holter recordings showed improvement in HRV of reduced ejection fraction in heart failure patients with BBs^33^ and in general HRV is sensitive to BB treatment for chronic heart failure including coronary artery disease and detects shifts in sympathetic with traditional time domain measures including RMSSD^35,36^.

However, the change in HRV measures caused by BB therapy for HFpEF patients have not been studied before. The aim of the current research was to investigate characteristics of cardiac rhythm in patients with preserved ejection function and the effectiveness of BB treatment over a 24-h period in order to clarify the rhythm characteristics that have the highest sensitivity to BBs and the time of day.

## Methods

### Dataset

The study included clinical datasets from American and Greek patients between the ages of 33 and 88 years (n = 303) and diagnosed with HF, more specifically CAD, of which 129 were classified into HFpEF (EF > 55%) according to the ASE/EACVI guidelines^37^.

Archives from University of Rochester Medical Center Telemetric and Holter ECG warehouse (THEW) of the Intercity Digital Electrocardiography (ECG) Alliance (IDEAL) were used to obtain American patient data^38,39^. The eligibility criteria to be included in the IDEAL study were as follows. (1) To have a history of MI or exercise induced ischemia; (2) After the last event, be in stable ischemia stage since the last 2 months, at least. (3) Have no diagnosis of a congenital heart failure. (4) Being in sinus rhythm. The exclusion criteria included individuals with malignancy diseases, non-sinus rhythm, any cerebral disease, coronary artery bypass grafting (CABG) surgery, congenital heart failure (CHF), severe hepatic disease and cardiomyopathy (left ventricular diameter (LVD) > 60 mm and EF < 40%).

The PRESERVE EF study was used to obtain data from seven cardiology departments in Greece^40^. The inclusion criteria for the study included: (1) To have a proven MI through post-angiography of either 90 days after any CABG surgery or at least 40 days after event. (2) Be revascularized (3) Not revascularized without any active ischemia in the past 6 months, and (4) Following medical therapy. The exclusion criteria of the study included individuals with persistent, long-standing and permanent atrial fibrillation, rheumatic diseases, cancer, any neurological symptoms of syncope or pre-syncope within the last 6 months, permanent pacemaker, any presence of any systemic illnesses such as liver failure, renal disease, thyroid dysfunction and a secondary prevention indication for implantable cardioverter defibrillator (ICD).

The exclusion criteria of the present study included patients taking medication other than BBs which included ACE-inhibitors, anti-arrhythmic and diuretic. Out of the total 73 patients for HFpEF, 56 were using beta-blockers (BBs) and 17 were not on beta-blocker medication (NBB). Table 1 represents the demographical data for each group.

**Table 1:** Demographic characteristics.

Characteristics	Preserved		
	With beta-blockers (BB, n = 56)	Without beta-blockers (NBB, n = 17)	*p* value
Age (years)	56.6 (12.8)	57.9 (10.5)	0.656
Gender (male/female)	47/9	15/2	0.005
BMI	26.5 (3.97)	25.7 (3.07)	0.256
LVEF	66.6 (5.75)	65.8 (7.45)	0.804
Mean heart rate	65.6 (8)	73.1 (9.59)	**0.0035**

Data are expressed as mean (SD) for continuous variables and n for categorical, *BMI* Body mass index (kg/m^2^), *LVEF* left ventricular ejection fraction(%).

Significant value are in bold.

### Heart rate variability feature extraction

HRV signal was calculated for each hour of the Holter ECG recordings using Pan Tompkins^41^ and pre-processed for noise using the SDROM-ADF filter^42^. Cosinor fitting analysis was used to fix the starting point of the 24-h circadian rhythm at 12:00 am and HRV features extracted.

Frequency-domain, time-domain, non-linear metrics and fragmentation indices were included in the HRV analysis. PhysioZoo open access toolbox was used to extract HRV features in Matlab 2022a (MathWorks)^43^. Average N-to-N intervals (AVNN), standard deviation of the N-to-N intervals (SDNN), root mean square of differences between successive N-to-N intervals (RMSSD) and percentage of successive RR intervals differing by more than 50 ms (pNN50) were included in the time-domain features which defines the interbeat interval variability^44^. Similarly, distribution of power is explained through the frequency-domain measures including discrete frequency bands which are high frequency, HF, 0.15–0.4 Hz, low frequency, LF, 0.04–0.15 Hz, and, very low frequency, VLF, 0.0033–0.04 Hz^44^. Sample entropy and fragmentation measures of PAS (percentage alternation segments), IALS (inverse average length of segments), PIP (percentage of inflection points in the N-to-N interval), and PSS (percentage of short segments) were also included^44,45^.

### Correcting for heart rate

Due to the nonlinear relationship between R–R interval (RRi) and heart rate (HR), the fluctuations of RRi are dependent on average HR^46–48^. Even when the variability of the heart does not change, a decrease in the average HR causes an increase in RRi oscillations, and vice-versa^48,49^. When the average HR changes, it causes an amplification effect of the ANS on the HRV. There is a significant association between HRV, and HR, hence, HRV provides information of both HR and its variability^50^. Therefore, it is challenging to determine which of the two, HR and its variability, plays a role in HRV prognostic value. The nonlinear relationship between RRi and HR causes a mathematical correlation between HRV and HR^51^. Sacha^50^ recommends that for HRV parameters which have a negative association with HR, the HRV parameters should be divided by the suitable power of average RRi to decrease the influence of HR on HRV indices. On the other hand, in case of a positive relationship between HRV parameters and HR, the parameters must be multiplied by appropriate power of average RRi to remove, both, the physiological and mathematical HRV dependence on HR^46,47,50,52^. The effects of dividing and multiplying the HRV parameters used in this study by different powers of average RRi was investigated and the Pearson correlation calculated for them. The power of RRi with the correlation closest to 0 was chosen to correct the HRV parameters.

### Statistical analysis

12 HRV features using 1-h, 1-h, and 8-h windows for standard and corrected HRV features were extracted to investigate the short-term and long-term effects of the BB therapy on HRV. Furthermore, day (06:00–18:00) and night (18:00–06:00) time HRV features for both groups were also studied.

To assess the normality of the data distribution, the Kolmogorov–Smirnov test was used. Statistical differences between the two groups for the HRV measures was determined using the nonparametric Mann–Whitney U test with significant p value set at less than 0.05. Common language effect size, c, is defined as:

1 $$c=\frac{{U}_{1}}{{n}_{1}{n}_{2}},$$

where U_1_ is the smaller Mann–Whitney test statistic, n_1_ is the sample size of group 1 and n_2_ is the sample size of group 2^53^.

In addition, the percentage change in HRV features (% $\Delta$ HRV_RBB_) of the BB group relative to the HRV features of NBB group was calculated as a measure of change between the two groups shown in Eq. (2).

2 $$\% {\Delta HRV}_{RBB}=\frac{{HRV}_{BB}-{HRV}_{NBB}}{{HRV}_{NBB}} \times 100,$$

where HRV_BB_ are the HRV features for BB group and the HRV_NBB_ refers to the HRV measures for NBB group.

Univariate and multiple regression analysis was carried out to identify the effect of the use of BBs on standard and corrected HRV parameters. The effect size was measured by calculating Cohen’s *f*^2^ within a multiple regression model^54^. The *f*^2^ were calculated to measure local effect size and combined effect size of the predicted model. According to Cohen’s guidelines, *f*^2^ ≥ 0.02, *f*^2^ ≥ 0.15, and *f*^2^ ≥ 0.35 represent small, medium, and large effect sizes, respectively^55^. All the analysis in the study was undertaken in Matlab 2022a (MathWorks).

### Ethics approval and consent to participate

Title 45, U.S. Code of Federal Regulations, part 46, protection of human subjects, and Declaration of Helsinki were followed for database enrollment of IDEAL database. The IDEAL protocol was approved by University of Rochester and informed consent was signed by all the patients. For the PRESERVE EF study ethics committee at each cardiology department approved the study which was validated by Hellenic Society of Cardiology. Informed consent was signed by the patients before enrollment at each respected cardiology department.

## Results

### Heart rate variability correction for heart rate

**Figure 1:**
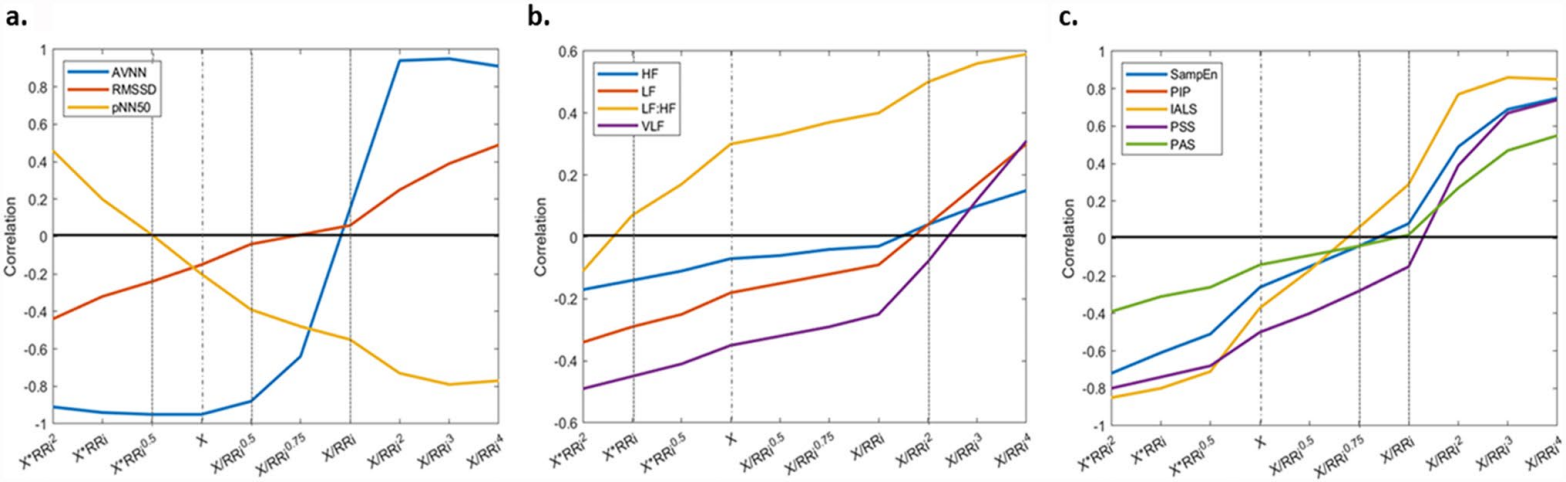
Correlation coefficients between the HRV indices and HR. Correlation coefficients between HRV features and HR are presented for standard and corrected HRV. The X denotes standard HRV indices which are all inversely associated with HR. Multiplication/division by different powers of the corresponding average RR intervals (RRi) were used to correct the standard HRV measures. The dotted lines show the correlation coefficient at X, the uncorrected HRV measures and at the corresponding power used to multiply/divide HRV indices to correct them. (**a**) The correlation coefficients for the time domain features AVNN, RMSSD and pNN50 are shown. (**b**) The correlation coefficients for the spectral indices of HRV HF, LF, LF:HF and VLF are presented. (**c**) The correlation coefficients for sample entropy and the fragmentation measures, PIP, IALS, PSS and PAS are presented.

Figure 1 shows the results for correction of the dependence of HRV on HR. All HRV measures except LF:HF ratio were found to have a negative correlation with average HR calculated for each hour. For all HRV measures except pNN50, multiplication by different powers of the corresponding average RR interval (RRi) increases this negative relationship as is shown in the left half of the graph and division by RRi to different powers reduces the dependence of HRV indices on HR and also inverse this relationship from negative to positive for higher powers as is demonstrated in the right half of the graph. The case of pNN50 is interesting as it demonstrates the opposite effect (Fig. 1a.) despite showing a negative relationship with HR. Multiplication with different powers of RRi reduces the negative relationship and then inverses it from negative to positive. However, insignificant correlations close to zero were not observed for all the HRV indices. Standard time domain measures lost their dependence on HR after dividing AVNN by RRi (p < 0.001), and RMSSD by RRi to the power of 0.5 (p = 0.07). pNN50 was multiplied by RRi to the power of 0.5 (p = 0.7). For spectral indices, HF (p = 0.1), LF (p = 0.13) and VLF (p = 0.001) were divided by RRi to the power of 2. LF:HF ratio was multiplied by RRi (p = 0.002). Sample entropy stopped being dependent on HR after dividing it by RRi to the power of 0.75 (p = 0.14). The fragmentation measures PIP (p = 0.01) and IALS (p = 0.01) were also divided by RRi to the power of 0.75. PSS (p < 0.001) and PAS (p = 0.46) were divided by RRi. Although, the correlation between the corrected HRV indices and average HR were very small but not all these relationships were insignificant.

### Heart rate variability analysis of different window size

This section will discuss the results of the analysis performed on 24-h ECG recordings to study the short- and long-term effects of BBs on HRV in HFpEF. This analysis was conducted using 1-h, 4-h and 8-h window. In addition, the HRV changes during day (06:00–18:00) and night (18:00–06:00) were also examined.

**Figure 2:**
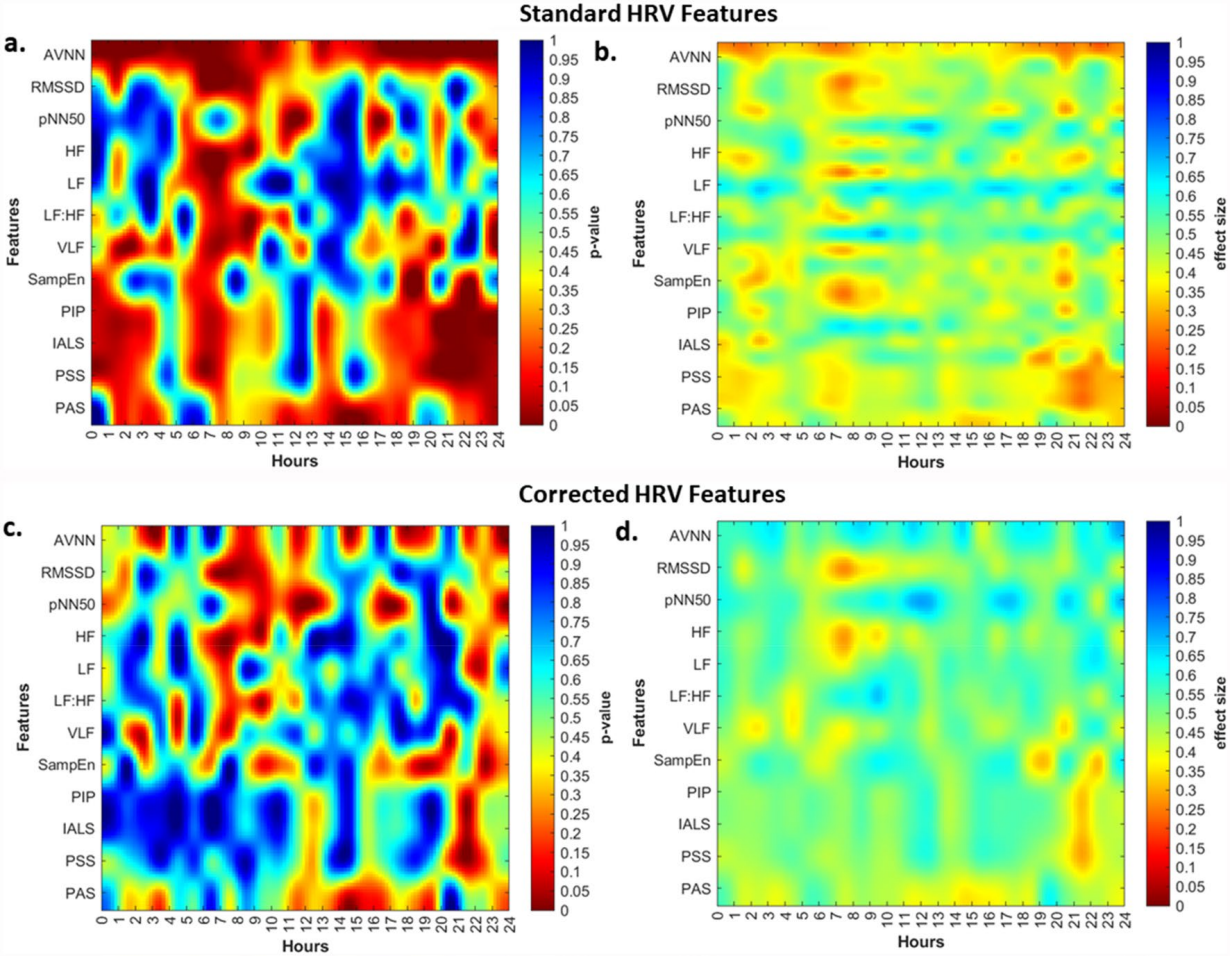
One-hour Window analysis for 24-h HRV Signal. Heat maps of p value and effect size for one-hour window analysis between BB and NBB group for HFpEF for standard and corrected HRV. (**a**) p values for standard HRV measure. (**b**) Effect size for standard HRV measures. (**c**) p values for corrected HRV measure. (**d**) Effect size for corrected HRV measures.

The results for the Mann–Whitney analysis with the effect size for 1-h window for the HFpEF are displayed in the Fig. 2.

Figure 2a,b display the p values and effect size for the standard HRV measures for HFpEF. AVNN was significantly different between the BB and NBB group for the hours of 00:00 to 04:00, 05:00 to 9:00 and 17:00 to 23:00 with moderate effect size. RMSSD and pNN50 were significant between the hours of 06:00 to 09:00 and 11:00 to 13:00 respectively. The effect size was moderate for both these time domain metrics. In addition, all spectral indices demonstrated difference between the two groups during different hours of the day. HF power has statistical significance during 06:00 to 08:00 and 09:00 to 10:00 while LF power is different between the BB and NBB group during 07:00 to 08:00 h. VLF power is significantly different for the following hours of the day, 02:00 to 03:00, 07:00 to 08:00 and 20:00 to 21:00. LF:HF ratio was only different during the hours of 08:00 to 09:00. The effect size for all spectral metrics investigated was small to moderate.

Furthermore, similar results for the spectral measures normalized by total power investigated for the 24 h using one-hour window were observed. Normalized HF (HFnu) was significantly different between the two groups for the morning hour of 07:00 to 08:00 (p = 0.045) and normalized LF (LFnu) showed significance during 02:00 to 03:00 (p = 0.021), 09:00 to 10:00 (p = 0.031), 16:00 to 17:00 (p = 0.032), 20:00 to 21:00 (p = 0.041) and 23:00 to 00:00 (p = 0.023). Normalized VLF (VLFnu) was only significant between 02:00 to 03:00 (p = 0.043). The hours of 18:00 to 20:00 and 22:00 to 23:00 were different between the two groups for sample entropy. The fragmentation measures of HRV namely PIP, IALS and PSS were statistically significant between the hours of 20:00 to 24:00 while PAS was only changed for the hours of 14:00 to 16:00.

Correcting HRV features for the dependence on average HR removes the significant changes observed in some hours of the day as is noted in Fig. 2c. AVNN is only significant for the hours of 23:00 to 24:00 while RMSSD shows difference in the morning at 07:00 to 08:00. Corrected pNN50 showed additional significant changes between the BB and NBB group for several hours of the evening and night. Corrected LF power, VLF power and PAS were not statistically significant for any hours of the day anymore. The change is detected in HF power and LF:HF ratio are now only limited to 07:00 to 08:00 and 09:00 to 10:00 h. Sample entropy on the other hand had similar results with one less hour as significant. The changes observed in other fragmentation measures are also limited to 21:00 to 22:00 h. It is interesting to note that while there were no significant changes (p > 0.05) observed between the standard and corrected HRV measures except for AVNN, strengthening or weaking the dependence of HRV metrics on HR can change prognostic outcomes.

**Figure 3:**
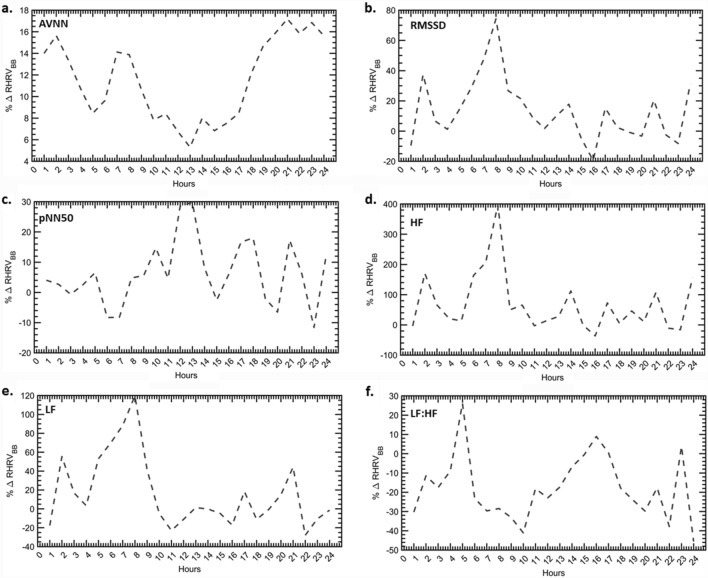
The relative percentage change in HRV features of the BB group relative to NBB for time and frequency domain measures. (**a**) AVNN. (**b**) RMSSD. (**c**) pNN50. (**d**) HF power. (**e**) LF power. (**f**) LF:HF ratio.

**Figure 4:**
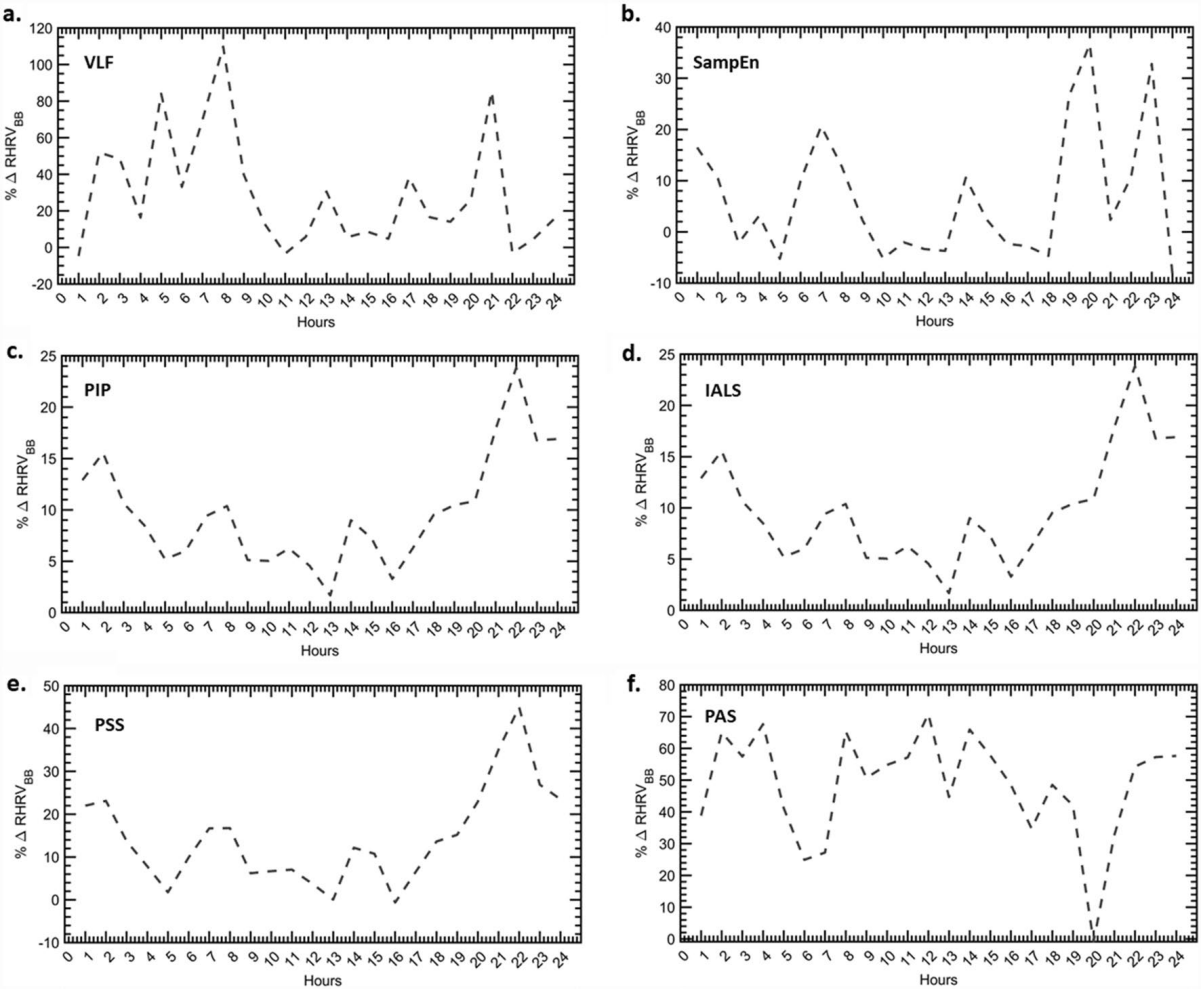
The relative percentage change in HRV features of the BB group relative to NBB for non-linear and fragmentation measures. (**a**) VLF. (**b**) Sample entropy. (**c**) PIP. (**d**) IALS. (**e**) PSS. (**f**) PAS.

The difference in HRV indices of the BB and NBB group relative to NBB group (% $\Delta$HRV_RBB_) was used as a measure of change in HRV metrics of the two groups for each hour. Figures 3 and 4 illustrate the comparative analysis of % $\Delta$HRV_RBB_ for HFpEF. Time domain features and spectral measures are higher in the BB group for most hours of the day. A significant increase in the morning hours for RMSSD, HF power and LF power is observed in the BB group. The LF:HF ratio indicates a decrease in the % $\Delta$HRV_RBB_ that is more prominent in the morning hours. Normalized spectral measures show similar profiles as the absolute powers. Sample entropy and all fragmentation measures show an increase for the BB group for most hours of the day_._

For the 4-h window analysis significant changes were only observed in AVNN for the following hours 00:00–04:00 (p = 0.006), 04:00–08:00 (p = 0.006) and 16:00–20:00 (p = 0.03) and 20:00–24:00 (p = 0.002). The fragmentation measures of PIP (p = 0.008), IALS (p = 0.008) and PSS (0.017) were statistically different between the BB and NBB groups for the evening hours of 20:00–24:00. The 8-h window shows significant changes for AVNN (00:00–08:00, p = 0.004 and 16:00–00:00, p = 0.005) PIP (16:00–00:00, p = 0.03) and IALS (16:00–00:00, p = 0.03) only.

The changes in % $\Delta$HRV_RBB_ throughout the day for 4-h and 8-h window analysis are illustrated in Table 2. All HRV measures except LF:HF ratio are increased for the BB group for most hours of the day. Time domain and frequency domain measures show a higher increase during the morning hours between 04:00 to 12:00 especially for RMSSD and HF power. LF power shows a decrease in the BB group from 08:00 to 16:00 and then during night from 20:00 to 24:00. A further analysis was conducted to understand the differences in HRV measures between the BB and NBB group for the hours of day, 06:00–18:00 and night, 18:00–06:00. No significant difference in the HRV measures between the two groups are detected.

**Table 2:** % ∆HRV_RBB_ for 4-h, 8-h and 12-h window analysis for all HRV measures.

	4-Hour window						8-Hour window				12-Hour window	
	0–4	4–8	8–12	12–16	16–20	20–24	0–8		8–16	16–24	Day (6–18)	Night (18–6)
AVNN	12.91	11.49	8.40	7.00	12.23	16.49	12.23		7.68	13.79	− 0.35	0.54
RMSSD	10.21	38.85	14.58	− 0.34	2.66	1.49	22.40		6.89	1.84	13.27	7.27
pNN50	0.97	− 1.63	12.83	9.47	7.68	1.69	− 0.06		11.24	6.49	2.44	0.24
HF	65.13	147.96	28.21	15.24	32.32	13.24	97.83		21.20	25.21	44.25	19.02
LF	14.02	80.32	− 3.20	− 6.22	3.92	− 5.64	40.50		− 4.72	− 0.90	13.53	2.57
LF:HF	− 17.41	− 17.85	− 28.82	− 4.38	− 17.25	− 24.18	− 17.11		− 17.70	− 21.27	− 3.59	− 3.59
VLF	23.60	70.85	12.21	10.80	23.66	14.04	43.76		11.56	20.01	4.27	0.88
SampEn	4.02	9.34	− 2.13	1.81	9.30	7.71	7.36		− 0.30	7.80	1.19	0.90
PIP	9.99	8.17	5.23	5.40	8.56	16.32	9.20		5.30	12.37	− 2.12	− 1.79
IALS	10.00	8.18	5.24	5.40	8.55	16.33	9.20		5.31	12.37	− 2.12	− 1.79
PSS	12.46	11.70	5.90	5.58	12.17	26.82	12.46		5.71	18.76	− 1.45	− 1.43
PAS	52.90	39.59	57.95	54.88	31.64	46.48	45.57		56.47	41.76	− 14.78	− 11.78

The % $\Delta$ HRV_RBB_ results in Table 3 shows that all HRV measures except LF:HF ratio and fragmentation measures are increased in the BB group. Changes in the spectral powers are higher during the day. LF:HF ratio does not show a change during the day and night period due to the drastic decrease of the LF power during the night. There was not much change in the AVNN and RMSSD value throughout the day in the BB and NBB groups.

**Table 3:** Univariate linear regression mode of the relation between beta-blocker therapy and heart rate variability features in preserved ejection fraction (HFpEF).

	β	*p*	R^2^	F-test
ANNN	0.0954	**< 0.001**	0.0743	138
RMSSD	0.00434	**0.0167**	0.00333	5.73
pNN50	− 0.0503	**0.0287**	0.00278	4.79
HF	190	**0.0158**	0.00339	5.84
LF	59.3	0.193	0.000989	1.7
LF:HF	− 0.609	**< 0.001**	0.01	17.3
VLF	361	**< 0.001**	0.00846	14.6
SampEn	0.0446	**0.0144**	0.00349	6.01
PIP	3.77	**< 0.001**	0.0296	52.4
IALS	0.0377	**< 0.001**	0.0297	52.5
PSS	5.03	**< 0.001**	0.0199	34.8
PAS	3.32	**< 0.001**	0.024	42.2

Significant values are in bold.

### Univariate and multivariate regression

To differentiate the possible effects of clinical variables and medical therapy separately or in combination on the effect of BB therapy on HRV measures in HFpEF univariate and multivariate linear regression analysis were performed. The results of the univariate analysis shown in Table 3 indicates that the use of BBs was associated with higher values of all time domain indices. Similarly, BB therapy indicated higher values for spectral indices except LF:HF ratio. However, the univariate model was not significant for LF power. Sample entropy and fragmentation measures were also increased.

A pairwise correlation among age, LVEF and HR was performed before the multivariate analysis. All variables were associated with each other except LVEF and HR. The correlations observed were weak and collinearity tests confirmed this finding. Table 4 demonstrates the results of the multivariate linear regression analysis for BB therapy when adjusted for age, LVEF and HR. A positive independent and significant relationship between BB therapy and higher HRV indices was only observed in AVNN and fragmentation measures PIP, IAL and PAS. After adjusting for clinical features, the independent positive relationship between the use of BB and HF power became of borderline significance. Whereas a negative relationship with borderline significance was observed in LF power and LF:HF ratio. Age and HR also significantly affected the outcome of HRV indices for most of the measures. Multivariate analysis was also performed for the corrected HRV feature adjusted for age and LVEF as is shown in Table 5. RMSSD shows borderline significance for the positive relationship with BB therapy. Time domain measure pNN50 shows decrease with the use of BB therapy for corrected HRV features too. In spectral indices, HF power and LF:HF ratio show significant models. Age and LVEF demonstrated significant contribution to the spectral indices for the standard and corrected HRV analysis.

**Table 4:** Multivariate linear regression model of the relation between BB therapy and standard heart rate variability features in preserved ejection fraction (HFpEF). *Bbuse* beta-blocker use (bold p values are significant).

		β	*p*	Local* f2*	Multiple R	p	*f2*
AVNN	Bbuse	0.00662	**0.018**	0.00332	0.902	**< 0.001**	9.17
	Age	0.000325	**0.00242**	0.00547			
	LVEF	− 0.000223	0.169	0.00112			
	HR	− 0.0117	**< 0.001**	8.02			
RMSSD	Bbuse	0.00281	0.134	0.00133	0.0523	**< 0.001**	0.0552
	Age	0.000444	**< 0.001**	0.0229			
	LVEF	− 0.000342	**0.00166**	0.00588			
	HR	− 0.000303	**< 0.001**	0.012			
pNN50	Bbuse	− 0.125	**< 0.001**	0.0168	0.0676	**< 0.001**	0.0725
	Age	− 0.0044	**< 0.001**	0.0142			
	LVEF	0.00207	0.129	0.00137			
	HR	− 0.00857	**< 0.001**	0.0607			
HF	Bbuse	160	0.0534	0.00222	0.0196	**< 0.001**	0.0199
	Age	11.9	**< 0.001**	0.00844			
	LVEF	− 7.51	0.118	0.00145			
	HR	− 6.84	**0.0221**	0.00311			
LF	Bbuse	− 85.8	0.0635	0.00204	0.0651	**< 0.001**	0.0696
	Age	− 11	**< 0.001**	0.0231			
	LVEF	− 6.13	**0.0223**	0.0031			
	HR	− 16.2	**< 0.001**	0.0557			
LF:HF	Bbuse	− 0.391	0.00576	0.00453	0.173	**< 0.001**	0.209
	Age	− 0.0792	**< 0.001**	0.128			
	LVEF	− 0.0122	0.138	0.00131			
	HR	0.04	**< 0.001**	0.0365			
VLF	Bbuse	− 122	0.18	0.00107	0.172	**< 0.001**	0.208
	Age	− 28.8	**< 0.001**	0.041			
	LVEF	− 9.13	0.0829	0.00178			
	HR	− 57.5	**< 0.001**	0.183			
SampEn	Bbuse	− 0.0397	**0.0257**	0.00296	0.14	**< 0.001**	0.163
	Age	− 0.00284	**< 0.001**	0.0103			
	LVEF	0.0011	0.307	0.000618			
	HR	− 0.0104	**< 0.001**	0.157			
PIP	Bbuse	1.41	**0.00144**	0.00604	0.372	**< 0.001**	0.529
	Age	0.259	**< 0.001**	0.139			
	LVEF	− 0.0115	0.655	0.000118			
	HR	− 0.362	**< 0.001**	0.304			
IALS	Bbuse	0.0141	**0.00144**	0.00604	0.372	**< 0.001**	0.593
	Age	0.00259	**< 0.001**	0.139			
	LVEF	− 0.000115	0.654	0.000119			
	HR	− 0.00362	**< 0.001**	0.304			
PSS	Bbuse	0.45	0.527	0.000237	0.388	**< 0.001**	0.633
	Age	0.326	**< 0.001**	0.0852			
	LVEF	− 0.0768	0.0629	0.00205			
	HR	− 0.68	**< 0.001**	0.415			
PAS	Bbuse	3.05	**< 0.001**	0.0214	0.136	**< 0.001**	0.157
	Age	0.251	**< 0.001**	0.0997			
	LVEF	0.103	**< 0.001**	0.0072			
	HR	− 0.0745	**< 0.001**	0.00981

**Table 5:** Multivariate linear regression mode of the relation between beta-blocker therapy and corrected heart rate variability features in preserved ejection fraction (HFpEF). *Bbuse* beta-blocker use (bold p values are significant).

		β	*p*	Local* f2*	Multiple R	p	*f2*
AVNN	Bbuse	1.78E−05	0.995	2.81E−08	0.00523	**0.0312**	0.00526
	Age	0.000234	**0.0214**	0.00314			
	LVEF	− 2.46E−04	0.117	0.00145			
RMSSD	Bbuse	0.00364	0.0567	0.00215	0.0523	**< 0.001**	0.0366
	Age	0.000483	**< 0.001**	0.0246			
	LVEF	− 0.000392	**< 0.001**	0.00681			
pNN50	Bbuse	− 0.108	**< 0.001**	0.015	0.0283	**< 0.001**	0.0291
	Age	− 0.00396	**< 0.001**	0.013			
	LVEF	0.00204	0.118	0.00145			
HF	Bbuse	198	**0.042**	0.00245	0.0128	**< 0.001**	0.013
	Age	10.5	**0.00601**	0.00448			
	LVEF	− 17.6	**0.00289**	0.00527			
LF	Bbuse	− 59	0.236	0.000831	0.0292	**< 0.001**	0.03
	Age	− 13	**< 0.001**	0.0263			
	LVEF	− 9.42	**0.00182**	0.00577			
LF:HF	Bbuse	− 0.364	**0.00128**	0.00616	0.129	**< 0.001**	0.148
	Age	− 0.069	**< 0.001**	0.144			
	LVEF	− 0.00976	0.154	0.00121			
VLF	Bbuse	52	0.569	0.000192	0.0369	**< 0.001**	0.0383
	Age	− 28.3	**< 0.001**	0.0369			
	LVEF	− 12.7	**0.0214**	0.00314			
SampEn	Bbuse	− 0.0315	0.087	0.00173	0.011	**< 0.001**	0.0112
	Age	− 0.00271	**< 0.001**	0.00833			
	LVEF	0.00124	0.267	0.000731			
PIP	Bbuse	0.704	0.126	0.00139	0.122	**< 0.001**	0.139
	Age	0.269	**< 0.001**	0.132			
	LVEF	− 0.0354	0.204	0.000956			
IALS	Bbuse	0.00706	0.125	0.0014	0.122	**< 0.001**	0.139
	Age	0.0027	**< 0.001**	0.132			
	LVEF	− 0.000354	0.204	0.000957			
PSS	Bbuse	1.41	0.062	0.00206	0.111	**< 0.001**	0.125
	Age	0.407	**< 0.001**	0.112			
	LVEF	− 0.117	**0.0107**	0.00387			
PAS	Bbuse	2.68	**< 0.001**	0.0173	0.112	**< 0.001**	0.126
	Age	0.265	**< 0.001**	0.11			
	LVEF	0.0929	**0.00195**	0.0057			

## Discussion

Heart failure with preserved ejection fraction is not a sole disease but refers to a range of heterogeneous pathophysiological conditions. Multiple therapeutic strategies have failed in showing a clear clinical improvement in HFpEF which demonstrates inadequate knowledge about the intricate and heterogeneous pathophysiology of this disease^56,57^. Furthermore, there is a lack of longitudinal studies to investigate potential treatments for the future^56^. Beta-blockers improve myocardial function and prolong survival in patients with heart failure. One mechanism of action of BBs is thought to be the interruption in the sympathetic nervous system^58–60^. However, studies also show the patients with HFpEF derive little or no survival benefit from the treatment with BBs^1,61^. Despite the controversial evidence BBs are regularly prescribed to > 70% of patients with HFpEF^62^. The use of BBs reduces the mean heart rate significantly^63^. In the present study significant changes in HR are observed for HFpEF.

Recent studies have indicated unfavorable hemodynamic effects of pharmacological lowering of HR within a subgroup of patients with normal ejection fraction^64–66^. Whereas increasing HR in a pacemaker study of patients with preserved ejection fraction within a physiological range has shown improvement in patients with HFpEF^63,64^. However, these studies are conducted on specific subsets of patients within HFpEF. It is worthwhile to note that BBs have been reported to improve functional performance of specific phenotypes of HFpEF such as those with atrial fibrillation and ischemic heart disease^62,64,65^. The lack of agreement on the effectiveness of BBs in HFpEF can therefore be ascribed to the clinical heterogeneity of this syndrome. Investigations on patients with HFpEF that were administered BBs have been inconsistently described and do not account for their specific phenotype or other critical factors such as gender, age, resting heart rate and presence of stable angina among other considerations that could influence the suitability of BB treatment^56,57,66^.

The main aim of this paper was to address whether BBs are effective in HFpEF from the perspective of the changes observed in the cardiac autonomic modulation following BBs administration using HRV as a feature to describe modulation of cardiac rhythm. It is crucial to emphasize that our analysis only pertained to a particular subset of HFpEF with ischemic heart disease, and therefore, these findings need to be further investigated in other HFpEF phenotypes.

HRV metrics are known to be influenced by the chronotropic state^46–48^, therefore the HRV measures for the one-hour window analysis were corrected. Correcting for HR did not change the results for most of the HRV measures. Previous studies have shown treatment with BBs improved the linear and nonlinear HRV measures in patients with reduced ejection fraction^57,67^.

A study investigating the effect of propranolol on the HRV measures following acute myocardial infraction found significant changes in the RMSSD measure^68^. The results of the current analysis confirm these findings for HFpEF. In another investigation on the effects of BB therapy in decompensated heart failure, the use of BB therapy increased RMSSD and pNN50, the two-time domain measures of parasympathetic cardiac activity, by 39% and 57% in the BB group respectively^63^. The present study shows similar results with an approximate 10% and 1.34% increase over 24 h in RMSSD and pNN50 respectively indicating an improvement in vagal modulation. Further, patients with coronary artery disease also demonstrated that administration of BBs significantly increased HF power and RMSSD^33^. Effects of propranolol in acute myocardial infarction patients showed a significant increase in HF power and a decrease in the LF:HF ratio, the latter is a measure of sympathovagal balance^68^.

The different hour window analysis used here reveals the greatest change in HRV measures between the two groups are observed between 04:00–12:00 and 18:00–00:00 h which coincides with the morning (06:00–10:00) sympathetic surge that is associated with an increased risk of an adverse cardiovascular event and a secondary smaller peak reported for the time period between 18:00–00:00, which is in agreement with previous studies^30,69–71^.

The impaired sympathovagal balance in heart failure patients can be observed by the decrease in the HF power and an increase in the LF frequency^1^. Spectral analysis of HRV for decompensated heart failure revealed that spectral powers were higher in patients with BBs although the difference was not statistically significant for VLF and LF power concurrent with the present work for HFpEF^63^. The results of this study exhibit an improved sympathovagal balance with significantly increased HF and LF power and consequently lowered LF/HF ratio for the BB group in comparison to the NBB patients for HFpEF. This may indicate a possible reduction in the sympathetic surge in the morning hours and a reduction in adverse cardiac event risk. The increase in short-term HRV measures including RMSSD and HF power seen in this study suggests the recovery of parasympathetic tone in HFpEF patients undergoing BB therapy.

A study that evaluated the sympathetic shift in heart failure patients on BB treatment through HRV measures found that the nonlinear HRV measure of sample entropy was not significantly affected^35^. Traditional time domain measures, such as RMSSD, were found to be better predictors of the sympathetic shift in heart failure^35^. The short-term fragmentation measures were found to be higher in patients with coronary artery disease and outperformed the traditional linear measures and sample entropy in separating healthy subjects from patients^45^. However, a decrease in the fragmentation measures was only observed in the day and night analysis analysis for BB group for HFpEF in this work. The advent of cardiovascular disease and old age has shown to produce rough variability patterns in the sinus rhythm that are not attributed to vagal tone modulation and can be computationally measured by the heart rate fragmentation^45^. This phenomenon can explain the increase in the fragmentation measures despite the decrease in short-term HRV measures of vagal modulation. The effect of BBs during the day and nighttime has also been previously reported, with greater effect during the day^72^. This pattern can also be observed in the current study especially for the frequency domain measures.

In addition, a positive relationship for HRV time domain measures and frequency domain measures of HF and LF power for the univariate and multivariate analysis adjusted for clinical and treatment characteristics have been reported^63^. The univariate analysis of the current study shows similar results but the model for LF power is not significant. However, the significant relationship between BB therapy and higher time domain measures is only seen in AVNN for adjusted models for standard HRV while pNN50 decreases with the use of BBs for models adjusted for age, LVEF and HR for both standard and corrected HRV.

A distinct relation between reduced HRV and the increased risk of mortality in cardiac patients has previously been reported^73,74^. Numerous clinical and experimental investigations have indicated that the risk of mortality related to ischemia is affected by changes in the parasympathetic activity^75–78^. In patients with chronic heart failure (CHF), decreased vagal activity is linked to higher mortality rates, and the withdrawal of vagal activity is an early indicator of acute decompensation^79,80^. Increase in the parasympathetic activity through chronic stimulation of the vagus nerve (VNS), pharmacologically or by exercise showed beneficial results and a significant improvement in the hemodynamics of the left ventricle and a decrease in mortality^75,75,81^.

Adrenergic overdrive is associated with an impaired baroreflex function in heart failure and is only observed in patients with reduced ejection fraction^5^. This means that a major mechanism regulating sympathetic function is affected differently in preserved and reduced groups. It is assumed that this difference is due to the severity of impairment of LVEF which is absent in HFpEF which could be one of the reasons of differences were observed in the changes in HRV for the two groups.

This study has potential limitations. Although this study presents an overview of the effects of BB therapy on HFpEF group, subject specific information about the type of BB agent taken and the duration of the therapy received by each subject was not available and should be taken into consideration for future work. Furthermore, data about the ethnicity and drug therapies for comorbidities for both groups of subjects was not recorded in the databases used and may influence the outcomes. Future studies in this field need to design longitudinal studies to investigate the clinical utility of BBs according to LVEF, type of BB agent, age, sex, ethnicity, HR and comorbidities in this patient population.

## Conclusion

The HRV analysis of patients with HFpEF receiving BB therapy demonstrated an overall increase in the HRV measures compared to the no medication group with significant differences in the HRV measures observed during the periods associated with high cardiac risk. This indicates towards an improvement in the cardiac autonomic regulation especially during these periods and suggests that BB therapy may be advantageous for this group of patients.

## Data Availability

The data used in this study is available upon request from the corresponding author.

## Abbreviations

ANS: Autonomic nervous system
BB: Beta-blocker
BBs: Beta-blockers
AVNN: Average of NN intervals
ECG: Electrocardiogram
HF: High frequency power
HFpEF: Heart failure with preserved ejection fraction
HFrEF: Heart failure with reduced ejection fraction
HR: Heart rate
HRV: Heart rate variability
IALS: Inverse average length of segments
LF: Low frequency
LVEF: Left ventricular ejection fraction
NBB: Without beta-blockers
PAS: Percentage alternation segments
PIP: Percentage of inflection points in the N-to-N interval
PNS: Parasympathetic nervous system
PSS: Percentage of short segments
RMSSD: Root means successive square difference
RRi: RR intervals
SampEn: Sample entropy
SEM: Standard error of the mean N-to-N interval
VLF: Very low frequency
